# Genomics of extreme ecological specialists: multiple convergent evolution but no genetic divergence between ecotypes of *Maculinea alcon* butterflies

**DOI:** 10.1038/s41598-017-12938-8

**Published:** 2017-10-23

**Authors:** Darina Koubínová, Vlad Dincă, Leonardo Dapporto, Raluca Vodă, Tomasz Suchan, Roger Vila, Nadir Alvarez

**Affiliations:** 1 Department of Ecology and Evolution, Faculty of Biology and Medicine, University of Lausanne, Biophore, 1015 Lausanne Switzerland; 2 Institut de Biologia Evolutiva (CSIC-Universitat Pompeu Fabra), Passeig Marítim de la Barceloneta, 37, 08003 Barcelona, Spain; 3 Department of Ecology and Genetics, University of Oulu, PO Box 3000, 90014 Oulu, Finland; 4 Department of Biology, University of Florence, via Madonna del Piano 6, 50019 Sesto Fiorentino, Florence Italy; 5 Dipartimento di Scienze della Vita e Biologia dei Sistemi, Università degli Studi di Torino, Via Accademia Albertina 13, 10123 Turin, Italy; 6 W. Szafer Institute of Botany, Polish Academy of Sciences, ul. Lubicz 46, 31-512 Kraków, Poland; 7 Natural History Museum of Geneva, Route de Malagnou 1, 1208 Geneva, Switzerland

## Abstract

Biotic interactions are often acknowledged as catalysers of genetic divergence and eventual explanation of processes driving species richness. We address the question, whether extreme ecological specialization is always associated with lineage sorting, by analysing polymorphisms in morphologically similar ecotypes of the myrmecophilous butterfly *Maculinea alcon*. The ecotypes occur in either hygric or xeric habitats, use different larval host plants and ant species, but no significant distinctive molecular traits have been revealed so far. We apply genome-wide RAD-sequencing to specimens originating from both habitats across Europe in order to get a view of the potential evolutionary processes at work. Our results confirm that genetic variation is mainly structured geographically but not ecologically — specimens from close localities are more related to each other than populations of each ecotype from distant localities. However, we found two loci for which the association with xeric versus hygric habitats is supported by segregating alleles, suggesting convergent evolution of habitat preference. Thus, ecological divergence between the forms probably does not represent an early stage of speciation, but may result from independent recurring adaptations involving few genes. We discuss the implications of these results for conservation and suggest preserving biotic interactions and main genetic clusters.

## Introduction

Biotic interactions represent essential components of ecosystems^1^. Ecological relationships such as host-parasite, resource-consumer, mutualism or competition, affect the realized ecological niche of species and, consequently, their reaction to habitat changes^2,3^. The impact of such changes should be higher in ecological specialists, rather than generalists, as specialists are usually more constrained by their specific biotic and abiotic requirements^4^. Strong interactions and more specific needs may thus lead to faster isolation of populations and development of reproductive barriers. The evolution of different ecotypes may be associated with genetic differentiation, catalyzing lineage divergence and eventually driving the speciation processes^5–8^.

Shifts in host-plant associations are found in many herbivorous insects, such as butterflies, in which it is often considered one of the main mechanisms producing their observed diversity^9–11^. However, in order to drive differential selection, the host-plant association character (most importantly adaptation to its defense secondary metabolites) should be inherited, variable, and conferring a local advantage. Whether or not host-plant association shifts occurred repeatedly within a species’ populations, or are associated with a deeper divergence of lineages, remains an open question in most herbivores showing multiple hosts^12,13^. So far, unraveling the evolutionary history of host-plant shifts has been rather limited due to the low resolution of classical genotyping and sequencing methods in a coalescent framework. However, with the advent of Next-Generation-Sequencing technologies, it is possible to analyse hundreds of loci from non-model species, and establish the extent to which host-plant association is linked with genetic differentiation, across the whole genome with a higher statistical power.

Here, we apply a genome-wide approach to address this longstanding matter for the highly specialized lycaenid butterfly *Maculinea alcon* (Denis & Schiffermüller, 1775), a «cuckoo» species of *Myrmica* ant colonies, feeding in the very early stages on a restricted number of plants and then imitating the ant brood and being fed by nurse ants. This strong ecological specialization potentially increases habitat patchiness and makes *Maculinea* a good ﻿model genus for the design of conservation strategies^14^, for understanding genetic variation structuring^15^, and for understanding the evolution of social parasitism^16^.

In Europe, *M. alcon sensu lato* is known to oviposit predominantly on three main larval host plants (*Gentiana cruciata*, *G. pneumonanthe*, and *Gentianella rhaetica*) and to parasitize at least five *Myrmica* ant species^17–19^ in contrasting habitats, hygric versus xeric, with few populations occupying a spatially restricted xeric habitat at high elevations. Some authors referred to the hygric ecotypes as “*pneumonanthe*” and to the xeric as “*cruciata*”, based on the host plant^20^; however, here, we assign the names based on their habitat^15,21^. Furthermore, to distinguish the low and high altitude xeric ecotypes, we refer to the populations at high xeric elevations as the “alpine“ ecotype. At the very few known syntopic localities (Răscruci, Romania and the Bükk Mountains, Hungary), the two low-elevation ecotypes also differ in their phenology, and their flight times only occasionally and partially overlap, limiting possibilities for gene flow^20,22–24^.

Two taxa were previously recognized within this system and often treated as different species: the hygrophilous *M. alcon* and the xerophylous *M. rebeli* (Hirschke, 1904), but see Kudrna and Frič^25^ vs. Tartally *et al*.^26^ for nomenclatural issues regarding the taxon *rebeli*.

It has been debated for nearly two decades whether these ecotypes are associated with genetic variation^20,27^. Moreover, adults lack unambiguous significantly segregating morphological traits^20,28^, so that individuals and populations are usually classified based on the larval host plant and locality type. Furthermore, the re-examination of *M*. *alcon* populations occurring at high altitude in the Alps revealed a series of differences compared to lowland populations—flight period (being likely caused by phenological differences associated with different elevations), larval host plants, ant hosts, wing pattern and genetic structure^20,25,26,29^— but these differences do not seem to justify the emergence of two distinct species^20^. Nevertheless, the different ecotypes and subspecies represent important evolutionary and conservation units^20^. We consider the existence of (at least) three ecotypes of *M. alcon* and provide the summary of their characteristics: 1) hygric (occurring in hygric habitats, feeding mainly on *G. pneumonanthe*, ovipositing mainly on the sepals of the flower buds and parasitising predominantly *My. rubra*, *My. ruginodis* and *My. scabrinodis*), 2) xeric (occurring in xeric localities up to 1,600 m, feeding mainly on *G. cruciata*, ovipositing predominantly on its leaves, and using as ant hosts mainly *My. schencki* and *My. sabuleti*), and 3) “alpine” (occurring at xeric localities above the coniferous tree level and, in the nominotypical population, feeding on *Gentianella rhaetica* and parasitising *My. sulcinodis* ^26,29–32^).

In addition to the differences mentioned above, the ecotypes can be distinguished by different flight periods (differing by one to a few weeks), which, however, vary slightly between the localities and years and are dependent on the flowering period of the host plant. Despite these ecological differences^33^, the morphological analyses revealed no or only very slight distinctive traits between the low-altitude ecotypes, with only the “alpine” ecotype differing by the wing pattern^20,28,29,33^. Similarly, classical molecular phylogenetic and population genetic studies from several localities of their area of occurrence, failed to reveal significant genetic differentiation between the low-altitude ecotypes^20,27,34,35^ despite their strong ecological differentiation. Minor ecotype-driven genetic differentiation in the few known syntopic/nearly syntopic populations was found, but it was not consistent with ecotype when other nearby populations were included^20,30,36^. The “alpine” population is usually referred to as «true *rebeli*». It shows a slight genetic differentiation and combined evidence based on a large number of phenotypic and genotypic markers suggested the existence of a subspecific differentiation^20^. The fact that no consistent genetic differences among the low-altitude ecotypes have been found so far does not necessarily mean they do not exist. Indeed, the current findings are based only on microsatellites, allozymes and classical sequencing of a reduced set of genes^20^. Theoretically, fine-grain differences could still be retrieved by performing genome-wide analyses. If differences among ecotypes are to be found, two hypotheses can be considered: (a) each of the forms is monophyletic and there is ongoing divergence between them, or (b) selection at a restricted number of loci occurred repeatedly with several independent evolutions of the ecotypes, indicating convergent evolution of habitat preference. Alternatively, if no genetic differences among ecotypes were found, even with a whole-genome sequencing approach, it would mean that the differences between the forms are likely caused by phenotypic plasticity or epigenetic variation only.

In order to test which scenario is at work in *M. alcon*, we investigated specimens of three ecotypes spanning the distribution of the species by applying Restriction site associated DNA sequencing (RAD-seq)^37^ and detecting single nucleotide polymorphisms (SNPs) at randomly distributed loci across the genome. We eventually discuss our results in light of conservation management.

## Results

The filtered dataset for 26 individuals (14 xeric, 11 hygric and one “alpine” ecotype – see Methods for detailed explanation) included 1,393 RAD loci. The SNP matrix is deposited in Zenodo ﻿(http://doi.org/10.5281/zenodo.997960)^38^ . In the additional dataset, where *Maculinea arion* was used as outgroup in order to identify the earliest diverging lineage in *M. alcon*, 949 RAD loci were retained. This dataset contained many loci that were monomorphic for *M. alcon* and would have produced a phylogeny with branch lengths equal or close to zero for all *M. alcon* specimens (due to shallow variability between them). After removing those monomorphic loci, 156 loci remained for the phylogenetic analysis.

### Population structure

**Figure 1:**
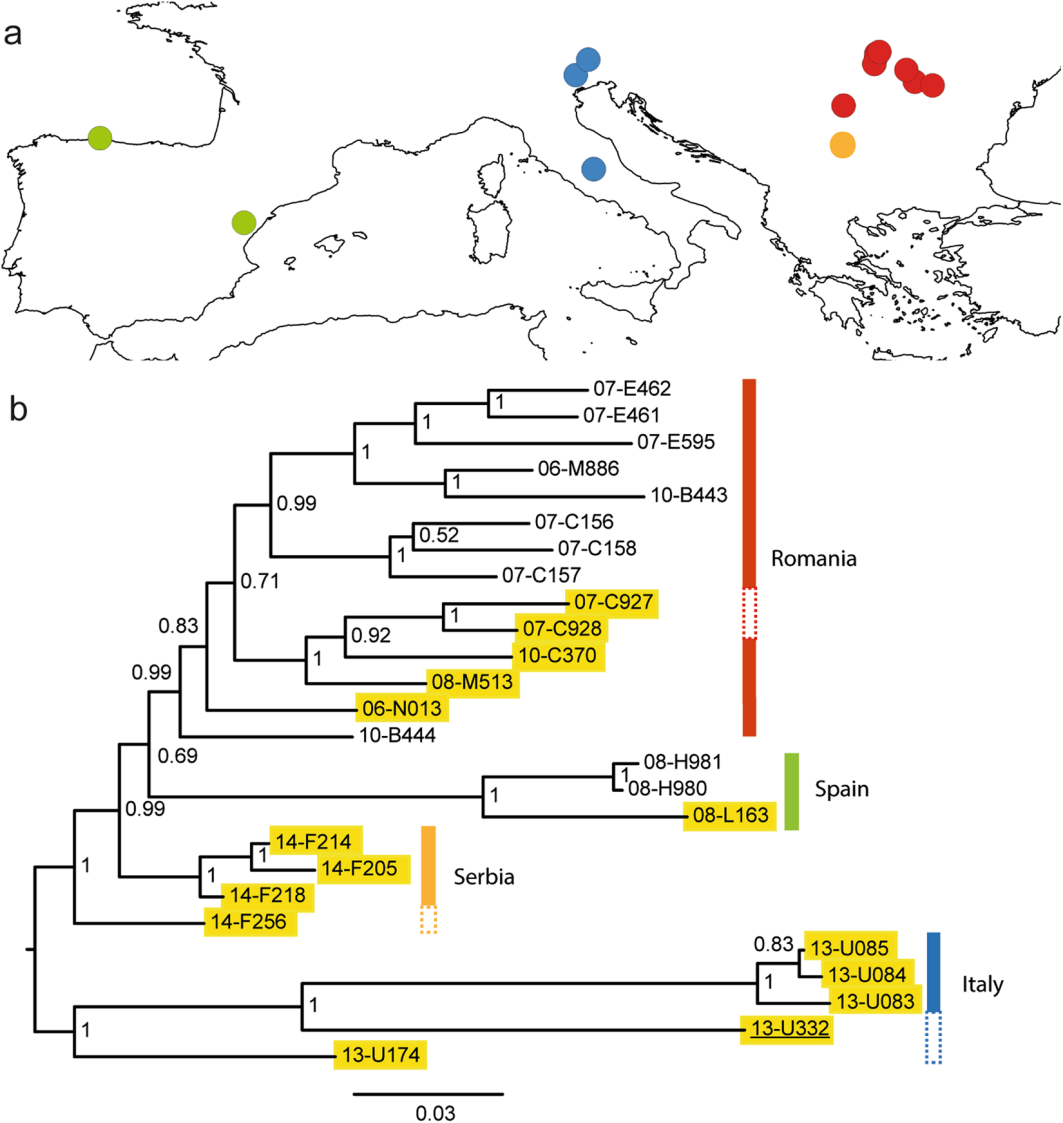
Sampling localities and phylogenetic tree analysis of 26 samples that belong to three ecotypes of *Maculinea alcon* (hygric, xeric and “alpine”): (**a**) Map of sampling localities of the *M. alcon* samples created in QGIS v.2.12.1; http://qgis.org; (**b**) Phylogenetic tree based on 1,393 SNPs constructed in PhyML. The xeric individuals are highlighted in yellow and the single specimen of the “alpine” ecotype is underlined. Node numbers indicate bootstrap support and branch length represents the number of substitutions. Colours in each figure (pie-charts in **a**, vertical bars in **b**, correspond to the STRUCTURE clusters, i.e. red – Romania, orange – Serbia, blue – Italy, green – Spain. Specimens demonstrating high levels of admixture and not assigned to specific STRUCTURE clusters are shown with a dashed vertical bar.

The Evanno method^39^ used to summarize the STRUCTURE outputs showed *K* = 4 as the most likely number of clusters. Three out of five runs for *K* = 4 showed the same pattern. We calculated mean cluster assignments from these three runs and used a threshold of 0.95 sample assignment to a given population (Table S1 and Fig. 1). A clear distinction among three xeric samples from central Italy (Monte Terminillo), three Spanish samples (two hygric and one xeric), three Serbian samples and 12 Romanian samples was observed. Five samples (all hygric, one from Spain, two from Romania and two from Italy) were characterized by large levels of admixture and could not be assigned to any group using the 0.95 assignment criterion.

### Phylogenetic analyses

The phylogenetic tree reconstructed with PhyML revealed four main clades, corresponding to the clusters detected by STRUCTURE: Spanish, Italian, Serbian and Romanian (Fig. 1). Hygric and xeric forms were not monophyletic, and only the geographical origin of samples, not ecotypes, was a structuring factor of the phylogeny. Also, the “alpine” sample clustered with the other Italian samples. The phylogenetic tree using *M. arion* as an outgroup showed much lower node support and poorly resolved clades, most likely a consequence of the matrix containing only 156 loci (data not shown). However, it allowed us to determine that the earliest diverging lineage within *M. alcon* was the Italian cluster (bootstrap support = 1).

### Correlation of genetic distances with geographical and ecological distances

The multiple regression on dissimilarity matrices explained nearly half of the overall variance (R^2^ = 0.492). Moreover, genetic distances were highly correlated with minimum path distance over land (regression coefficient = 0.700, p = 0.001) while they did not show any correlation with habitat type (regression coefficient = −0.020, p = 0.586).

### Loci putatively under selection and *Wolbachia* infection

**Figure 2:**
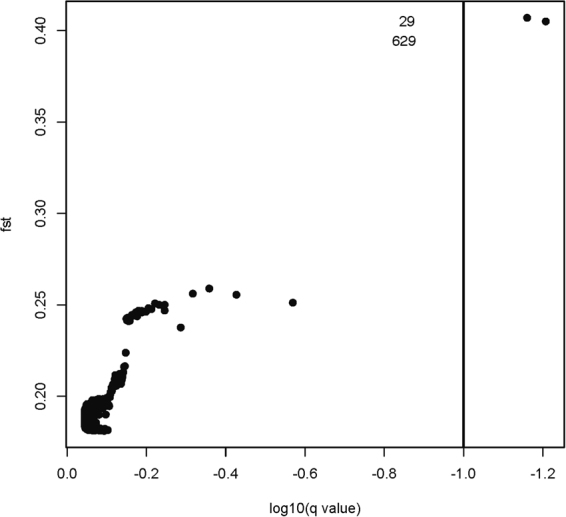
BayeScan output plotted in R. Two outliers (SNP 29 and 629) show a clear association with *M. alcon* ecotypes and are potentially under selection (FDR = 0.10).

The BayeScan analysis revealed two significant SNP outliers suggesting the existence of ecotype-associated selection at those loci (false discovery rate, FDR = 0.07–0.10; loci no 29 and 629 in the dataset; Fig. 2, data shown for FDR = 0.10; Table S2). Exclusion of the “alpine” ecotype produced identical results. A detailed examination of sequence composition revealed that the match was stricter for SNP 29, with all hygric forms bearing a T (homozygous or heterozygous with G) and all xeric forms bearing a G (homozygous or heterozygous with T). For SNP 629, we found two mismatches (i.e., two xeric individuals bearing a homozygote G, instead of A, or A/G, as observed in the rest of xeric specimens; Table 1). When blasting these two contigs using the *blastn* tool against lepidopteran sequences^40^, hits against butterfly mRNAs were found: for sequence 29, *blastn* retrieved a mRNA sequence coding for RING finger and SPRY domain-containing protein 1-like from *Papilio polytes* (XM_013291785.1), as well as an mRNA coding for elongator complex protein 1 from *Papilio machaon* (XM_014513286.1); for sequence 629, *blastn* retrieved an anonymous mRNA from *Bombyx mori* (AK405939.1) as well as a putative uncharacterized protein from *Papilio xuthus* (XM_013315886.1).

**Table 1:** Alleles distribution for the two loci potentially under selection in the three ecotypes of *M. alcon*, as indicated by the BayeScan analysis.

**Locus**	**29**			**629**		
Allele	G	T	G/T	G	A	G/A
Hygric	0	7	4	10	0	1
Low-altitude xeric	12	0	2	2	9	3
High-altitude xeric (“alpine﻿”)	1	0	0	0	1	0

Nucleotide variants occurring at each locus are summarized for all three ecotypes.

*Wolbachia* infection was found in all analyzed samples—32 among 2,116 contigs were of *Wolbachia* origin. For all 32 contigs, there was only one single allele in all analyzed specimens.

## Discussion

In this study, we investigated the genetic coherence of three ecotypes of the emblematic butterfly *Maculinea alcon*, using Next-Generation-Sequencing methods. Contrary to previous studies, applying classical population genetics methods to a local-scale sampling encompassing two ecotypes, we used whole-genome RAD-seq and investigated the ecological genomics of three ecotypes at a large geographical scale. Furthermore, we applied BayeScan to search for loci under selection. Similarly to previous studies^20,27,30,34–36^, our genome-level SNP analyses did not reveal strong genetic differentiation between the three ecotypes but mainly geographical structuring (see Fig. 1). This suggests that ecological shifts within this species are not associated with lineage sorting and do not represent an early stage of speciation, in contrast to processes occurring in many butterflies and other herbivorous arthropods^41–43^. Our result is in agreement with previous analyses based on spatially more restricted datasets of *M. alcon*, showing no significant genetic structure associated with the respective ecotypes and higher local genetic relatedness of the two low-altitude ecotypes of *M. alcon*, including syntopically occurring forms, than of populations of each ecotype from geographically distant localities^30,35,44,45^. Similarly, two individuals from our study (one hygric - 10-B443 and one xeric - 10-C370), originating from the same locality as studied by Tartally *et al*.^30^ did not cluster together, also suggesting a mixed origin of this population. Such a pattern is paralleled by observations in other species of the genus *Maculinea*, as for instance in *M. arion*, in which the two phenological forms differ in morphology and ecology but not genetically^46^.

Initially, it was thought that *Maculinea* species or forms depend on a single host ant and it was suggested that social parasitism and associated specific life cycle may chiefly affect the evolution, speciation and genetic background of these butterflies^32^. However, it has been subsequently shown that the usage of ant hosts may differ among localities, and may be adapted to exploiting multiple species or be switched to other species when the preferred host is not present or occurs only in low densities^17^. Therefore, the hypothesis regarding the influence of the ant host was revisited and some authors suggested that adaptation to the host plant (and corresponding habitat) is more likely to drive the evolution of *Maculinea* than would do adaptation to the ant species^30,46,47^. The host plants indeed represent allochronic resources as they usually flower at different times of the year^24^, because of the microclimatic conditions at the respective xeric and hygric habitats.

Our genome-wide study suggests that hygric and xeric ecotypes are statistically associated with different alleles at at least two loci (29 and 629). In one of the loci (29), homology to two types of mRNAs of the family Papilionidae (related to *Maculinea/*Lycaenidae), was established: (1) the RING finger and SPRY domain-containing protein is associated with oogenesis in Lepidoptera^48^ and (2) the elongator complex protein 1 is likely involved in acetylation of histones^49^ — although this has not been confirmed in invertebrates — and is recognized as a fast-evolving gene in insects^50^. Ecological differences in occupied biotopes, as well as in host plant phenology may explain the association with discrete differences in the oogenesis process. Overall, our results may be viewed as the footprint of multiple independent evolution of the two ecotypes, involving a few genes only. Similarly to Bereczki *et al*.^20^, we rule out a possible effect of *Wolbachia* infections in the segregation of ecotypes, given that all the samples analyzed were infected by the same bacterial strain.

The evolutionary history of the third ecotype found in an alpine habitat cannot be retrieved from our data, although the fact that it clusters with geographically close populations from Italy may suggest a similar process of recurring evolution. Interestingly, a recent study^20^ found that specimens from the Styrian Alps (the area from where the “alpine” ecotype has been described) are slightly genetically differentiated from the other populations of *M. alcon* analyzed. Further investigation including more specimens from this ecotype is needed to identify putative genetic differentiation between this and other ecotypes of *M. alcon*.

Several studies suggested the need to focus conservation efforts separately for each ecotype (despite the fact that, at least the low altitude ones, do not fulfill the criterion of evolutionary significant units), and indicated a higher vulnerability of the alpine and xeric ecotypes^26,30,36^. Although we did not find evidence for lineage sorting or early-stage speciation, we found at least two statistically associated markers for the hygric and xeric ecotypes, at least at the scale of the current study. Ecotypic distinctiveness is therefore associated with population-level evolutionary processes rather than with ongoing divergence. This case raises the question how to treat the intra-specific variation from a conservation perspective. The massive use of molecular methods in the last decades enabled researchers to find even minor genetic variations within species or populations, but it also triggered questions concerning the minimal genetic variation and minimal taxonomic unit that should be considered to define conservation units^51,52^. Since the 1990s the idea that even intraspecific variation should be considered for conservation gained more and more support^53^. However, levels of within-species genetic differentiation should not be the only criterion used when planning conservation strategies^54^. Although the *M. alcon* ecotypes may only differ at few loci, they each have a different ecological strategy and very specific interactions with other species, and thus they may likely differ in the factors affecting their survival, the adequate management, and the impact of their potential extinction in the ecosystems.

**Figure 3:**
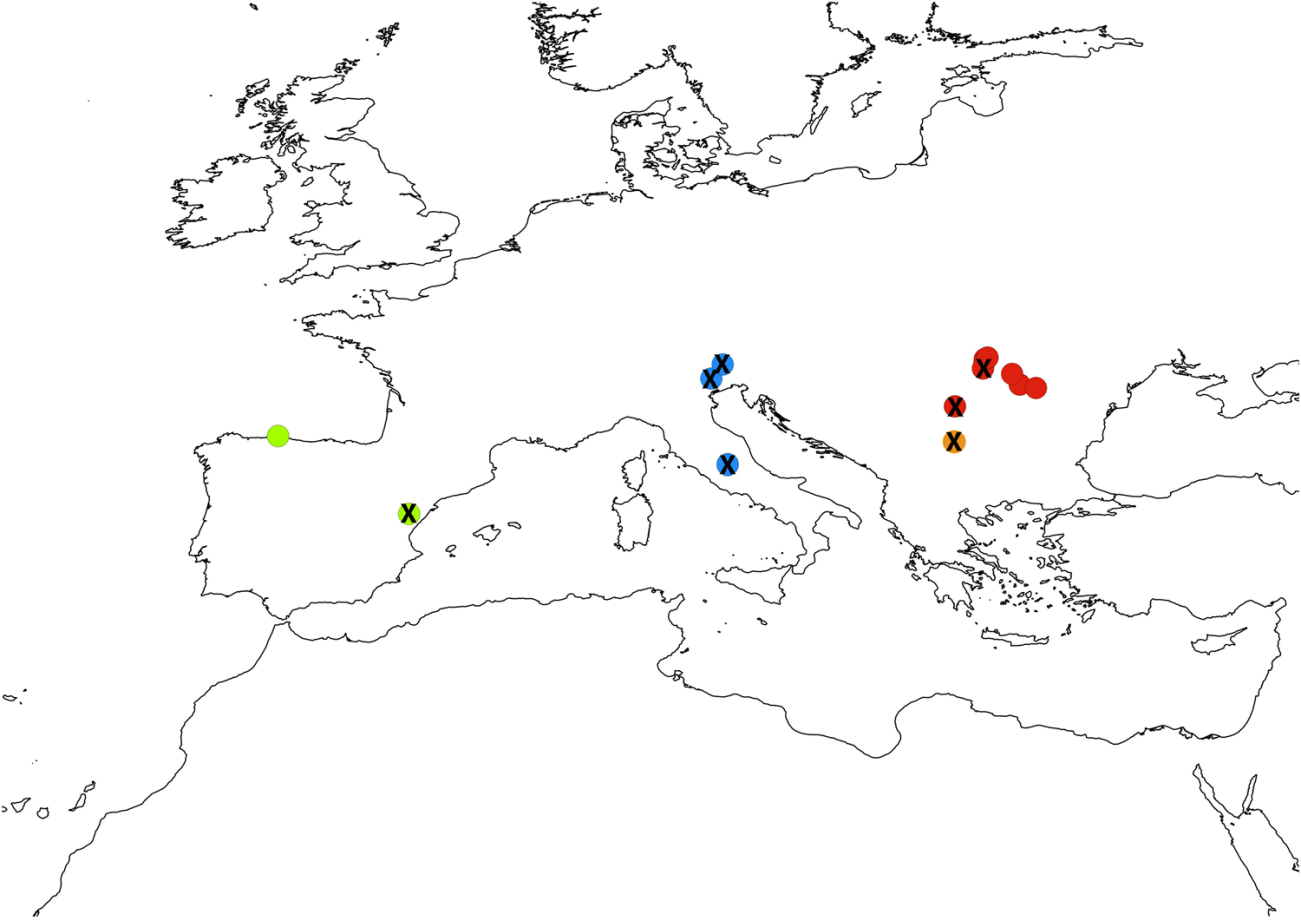
Schematic view illustrating optimal conservation focus in *M. alcon*, targeting ecotypes as well as genetic clusters. Colours indicate the four main lineages (as in Fig. 1), and an X indicates a population from xeric habitat. Only by protecting lineages and ecotypes (and thus associated biotic interaction), we can conserve both genetic and ecological processes. For simplicity we included only the hygric and xeric ecotypes. The map was created in QGIS v.2.12.1; http://qgis.org.

*Maculinea* species have become a model group not only for the study of parasitism and for insect conservation but also for preserving biotic interactions^55^, and we strongly support this view. Accordingly, we advocate a strategy aiming at conserving these ecotypes (each associated with specific biotic interactions) as well as the main genetic clusters (four detected in our study) despite the extremely low divergence within the species, this approach being the sole that guarantees that all diversity is protected (see Fig. 3). Moreover, we suggest further mapping of all the potential host ants and plants for these ecotypes, as gaining additional knowledge is crucial for appropriate conservation strategies. Additionally, experiments testing survival rates on non-preferred host plants (and ants) are key to test if natural selection could actually be generating divergence between ecotypes. Similarly, a wider sampling of ecotypes using all the potential variety of host ants (especially the so far undersampled populations using *My. rubra* and *My. ruginodis*) would be interesting to further confirm that there is actually no significant genetic divergence driven by the ant host specificity.

## Methods

### Field sampling

**Figure 4:**
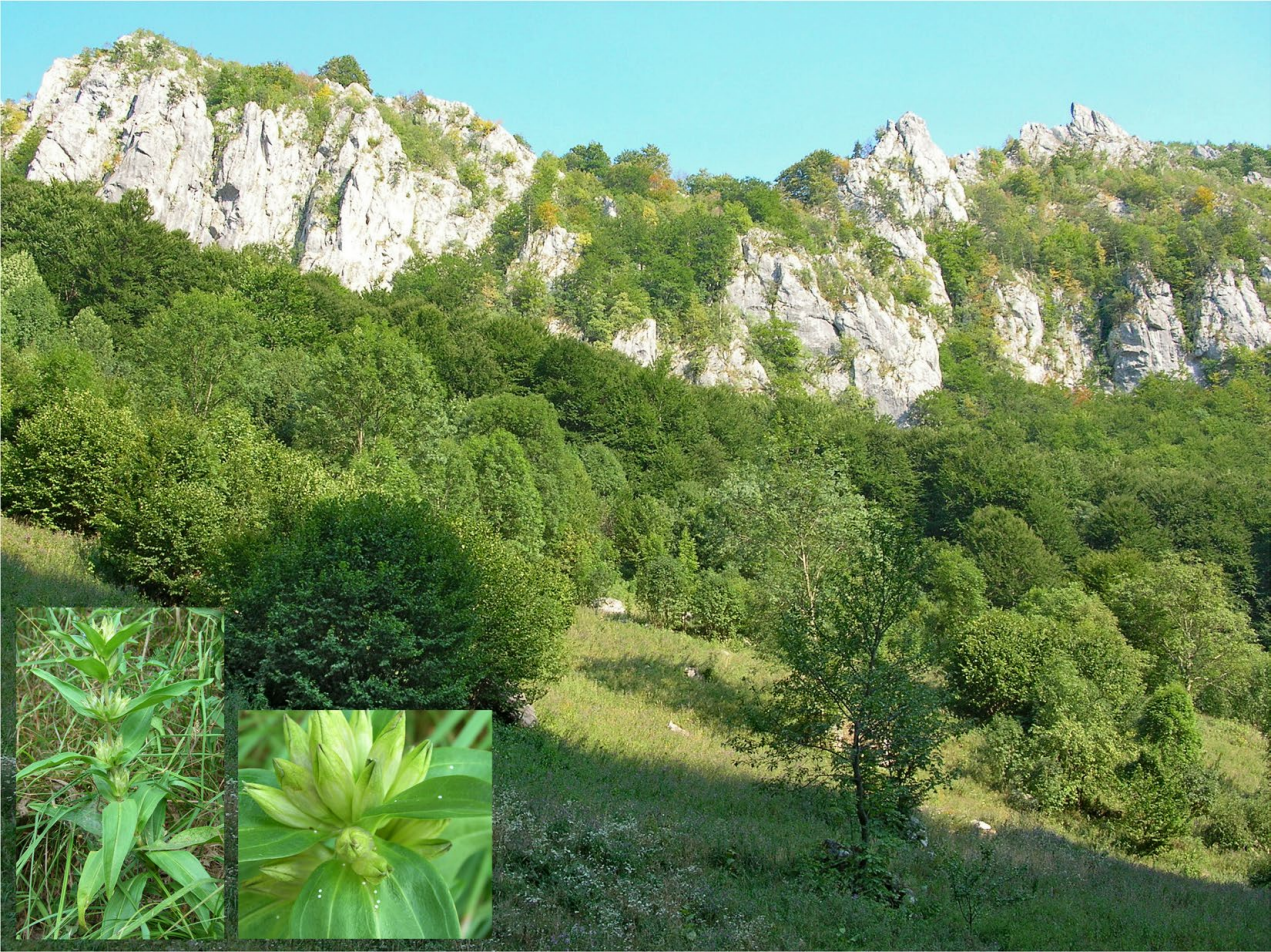
Low-altitude xeric habitat. The inset images show the host plant *Gentiana cruciata* (left) and its detail with *M. alcon* eggs (right). Locality: Romania, Caraș-Severin county, near Dobraia, 29. 7. 2007. Photo: Vlad Dincă.

**Figure 5:**
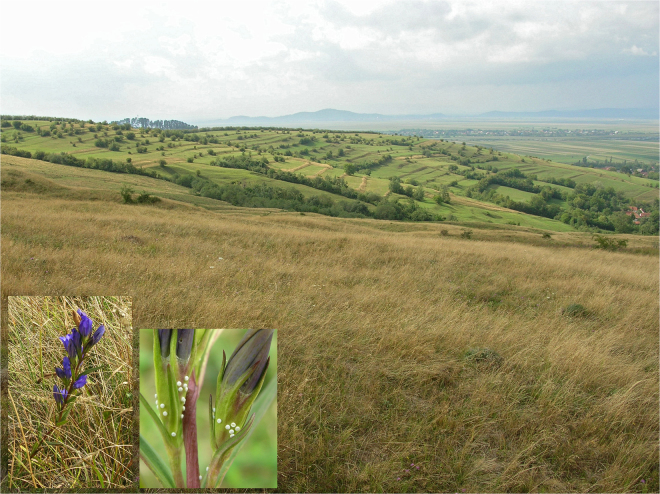
Hygric habitat. The inset images show the host plant *Gentiana pneumonanthe* (left) and its detail with *M. alcon* eggs (right). Romania, Brașov county, near Purcăreni, 14. 8. 2007. Photo: Vlad Dincă.

Twenty-six samples of *M. alcon* were collected from localities in Spain (3 individuals), Italy (5 individuals), Romania (14 individuals) and Serbia (4 individuals) between 2006 and 2014 (Table S3). The elevation of the localities ranged from 50 to ca. 1,800 m a.s.l. Fourteen specimens belonged to the xeric ecotype, at sites below 1,400 m a.s.l. where the host plant *G. cruciata* occurred (Fig. 4), 11 to the hygric ecotype at sites below 800 m a.s.l. where the host plant *G. pneumonanthe* occurred (Fig. 5), and one specimen was tentatively assigned to the “alpine” ecotype (collected at 1,817 m a.s.l. in Monte Chiadenis, Italian Carnic Alps, where the potential host plant *Gentianella* is present). However, without further study of more individuals from this latter population and comparison to the nominotypic alpine populations of the taxon *rebeli*, we cannot ascertain that our high-altitude sample belongs to this taxon and thus conservatively denominate it as “alpine” ecotype. One hygric and one xeric sample originated from a well-known locality near Răscruci, Romania, where both ecotypes are syntopic (Fig.  S1).

Adult individuals were captured using insect nets and stored in 98% ethanol at −20 °C. The field sampling was done under permits from the relevant authorities. All individuals are stored in the Butterfly Diversity and Evolution Lab at the Institute of Evolutionary Biology (CSIC-UPF) in Barcelona, Spain.

### DNA extractions and RAD-sequencing

#### Extractions

For most of the samples, whole genomic DNA was extracted from the thorax or abdomen, using BioSprint 96 robot with BioSprint 96 DNA Blood Kit (Qiagen, Hilden, Germany). The samples for which only legs were available (because other parts of the body had already been used for other studies) were extracted using the DNeasy Blood & Tissue Kit (Qiagen), in order to maximise the DNA yield. Obtained DNA isolates were diluted or concentrated to reach a final range of 20–50 ng/ul, based on measurements obtained with the PicoGreen dsDNA Assay kit (Thermo Fischer Scientific, Waltham, MA, USA) and FLUOstar Galaxy fluorescence microplate reader (BMG Labtech, Offenburg, Germany).

#### RAD-library preparation

The RAD-seq library preparation was performed using the double-digestion RAD protocol^37^, with slight modifications. The whole genomic DNA was digested in 9 ul reaction with 1 U MseI (New England Biolabs-NEB, Ipswich, MA, USA) and 2 U SbfI-HF (NEB) restriction enzymes, 1x CutSmart buffer (NEB) and 6 ul of genomic DNA, at 37 °C for 3 hours. Single strand adapter oligonucleotides were annealed by heating to 95 °C and slow gradual cooling. Adapter ligation was performed at 16 °C for 3 hours in a 20 ul reaction comprising 9 ul of the digested DNA, 0.5 uM of both RAD-P1 and RAD-P2 (Table S4) adapters, 1x T4 ligase buffer and 400 U of T4 DNA ligase (NEB). The reaction products were purified using AMPure XP (Beckman Coulter, Brea, USA), with a reaction/AMPure ratio of 0.8^56^. The purified template DNA was amplified by PCR (denaturation at 98 °C for 30 s, 13 cycles of 98 °C for 20 s, 60 °C for 30 s, 72 °C for 40 s and final extension at 72 °C for 10 minutes). The 10 ul reaction mix contained 1 ul of adaptor-ligated DNA, 0.2 U Q5 Hot-start polymerase, 1x Q5 buffer (both NEB), 0.2 mM of each dNTP, 0.6 uM of each Illumina primer (Table S4). Results of the reaction were verified using standard agarose gel electrophoresis. Samples were pooled and purified using the same protocol as before, with the AMPure ratio of 1.0. Fragments with a length ranging from 300 to 400 bp were selected using the Pippin Prep electrophoresis platform (Sage Science, Beverly, USA) and verified using Fragment Analyzer (Advanced Analytical). Libraries were sequenced on one lane of Illumina HiSeq using single-end protocol, at the Lausanne Genomic Technologies Facility (LGTF).

### DNA assembly and SNP calling

Raw sequence reads were demultiplexed using the process_radtags program^57^ and assembled *de novo* following the dDocent.FB pipeline (v.2.2.16)^58^. Final SNP data sets were filtered according to the dDocent filtering tutorial^59^: i.e. all loci present in less than 50% of the individuals, with Phred quality lower than 30 and minimum depth for a genotype call lower than 3, as well as samples having more than 90% of missing data, were removed. Minor allele frequency and allele balance at heterozygous sites filtering was applied. Additionally, we kept only biallelic loci, and discarded loci with missing information in more than 3 samples and all samples with more than 3 missing loci.

### Phylogenetic analyses and population structure

PGD Spider v. 2.1.0.3^60^ was used to convert the resulting VCF file to other formats. Phylogenetic analyses were performed with PhyML v. 3.3.2.0^61^ with the following changes to the default settings: GTR substitution model, estimated base frequencies, best of NNI and SPR tree topology search. Two runs, one without outgroup and one with four samples of *M. arion* as outgroup were performed. The *M. arion* samples, collected in the same way as the *M. alcon* samples, are deposited at the same institution and the same protocols were used for library preparation and calling (in addition to the previous filtering steps, the HWE filter was used).

The genetic clustering of individual genotypes was assessed using the Structure software version 2.3.4^62^, with the following settings: *K* = 2–10, burn-in = 100,000; number of MCMC repetitions after burn-in = 2,000,000; admixture model, 5 runs for each *K*. The results were visualized in Pophelper v1.0.10^63^.

### Correlation of genetic distances with geographical and ecological distances

Genetic distances among specimens were calculated in R v. 3.3.1^64^ within RStudio v. 0.99.903^65^ using Provesti’s distance as implemented in the poppr package v. 2.2.1^66^. A binary distance matrix was calculated for the ecological distance, attributing 0 to pairs of specimens belonging to the same ecotype, and 1 to those from different ecotypes. A geographical distance matrix encompassing the minimum path distance among specimens over land was produced—the minimum path distance was calculated by using the costDistance function as implemented in the gdistance R package (v. 1.2-1)^67^. The relative contribution of ecological and minimum path distances in explaining overall genetic distances was tested by multiple regression of dissimilarity matrix by using the MRM function implemented in the ecodist R package (v. 1.2.2)^68^. Since the variables were not normally distributed and the relationships not necessarily linear, a Spearman correlation was applied. *P*-values were calculated based on 1000 permutations.

### Loci putatively under selection and detection of *Wolbachia* bacteria

Candidate loci diagnostic of ecotypes were investigated with BayeScan v. 2.1^41^. The dataset was divided into three groups according to the three forms (xeric, “alpine”, and hygric) and two runs including and excluding the single “alpine” sample were performed. The results were plotted in R v. 3.3.1^64^ using the script plot_R.r implemented in BayeScan. FDR 0.5–0.10 was used, as the 0.10 threshold for *q*-values in BayeScan is more stringent than the same threshold for *p*-values in classical statistics^41^.

Additionally, because *Maculinea* butterflies are very often infected by *Wolbachia* bacteria, which is known to manipulate reproduction and to affect genetic variation structuring^69,70^, contigs obtained by dDocent for all specimens were blasted against available *Wolbachia* sequence data deposited in GenBank with the *blastn* tool^40^.

### Data Availability

The datasets supporting the conclusions are available in Zenodo (http://doi.org/10.5281/zenodo.997960).

## Electronic supplementary material

Supplementary Information

Table S1

Table S2

Table S3

Table S4
