# Supplementary material for: Genomics of extreme ecological specialists: multiple convergent evolution but no genetic divergence between ecotypes of Maculinea alcon butterflies
Source: Sci Rep. 2017 Oct 23;7:13752. doi: 10.1038/s41598-017-12938-8 (PMC5653870; doi:10.1038/s41598-017-12938-8)

Genomics of extreme ecological specialists: multiple convergent evolution, but no genetic divergence between ecotypes of *Maculinea alcon* butterflies

Darina Koubínová[1], Vlad Dincă[2], Leonardo Dapporto[2,3], Raluca Vodă[4], Tomasz Suchan[1,5], Roger Vila*[2], Nadir Alvarez*[1]

Supplementary information

Table S1 Detailed STRUCTURE results for K=4, mean assignment probability of three among five runs showing convergent pattern. Colours show clearly defined clusters with a cut-off threshold of 0.95. Samples with mean cluster assignment probability not reaching this threshold were considered as not assigned to any cluster. Colours of each cluster are identical as in Figure 1. Only sampling country and ecotype are mentioned for each individual, for precise information see Table S2.

Table S2 Detailed information about the two SNP outliers, revealed by BayeScan.

Table S3 Specimens and sampling localities of *Maculinea alcon* and *M. arion* used in this study. The specimens of both low-altitude ecotypes sampled syntopically, are highlighted in yellow.

Table S4 Adaptors and primers used in this study.

Figure S1 Locality in Răscruci, Romania, where both low-altitude ecotypes of *M. alcon* are syntopic. Photo Raluca Vodă, 2009.

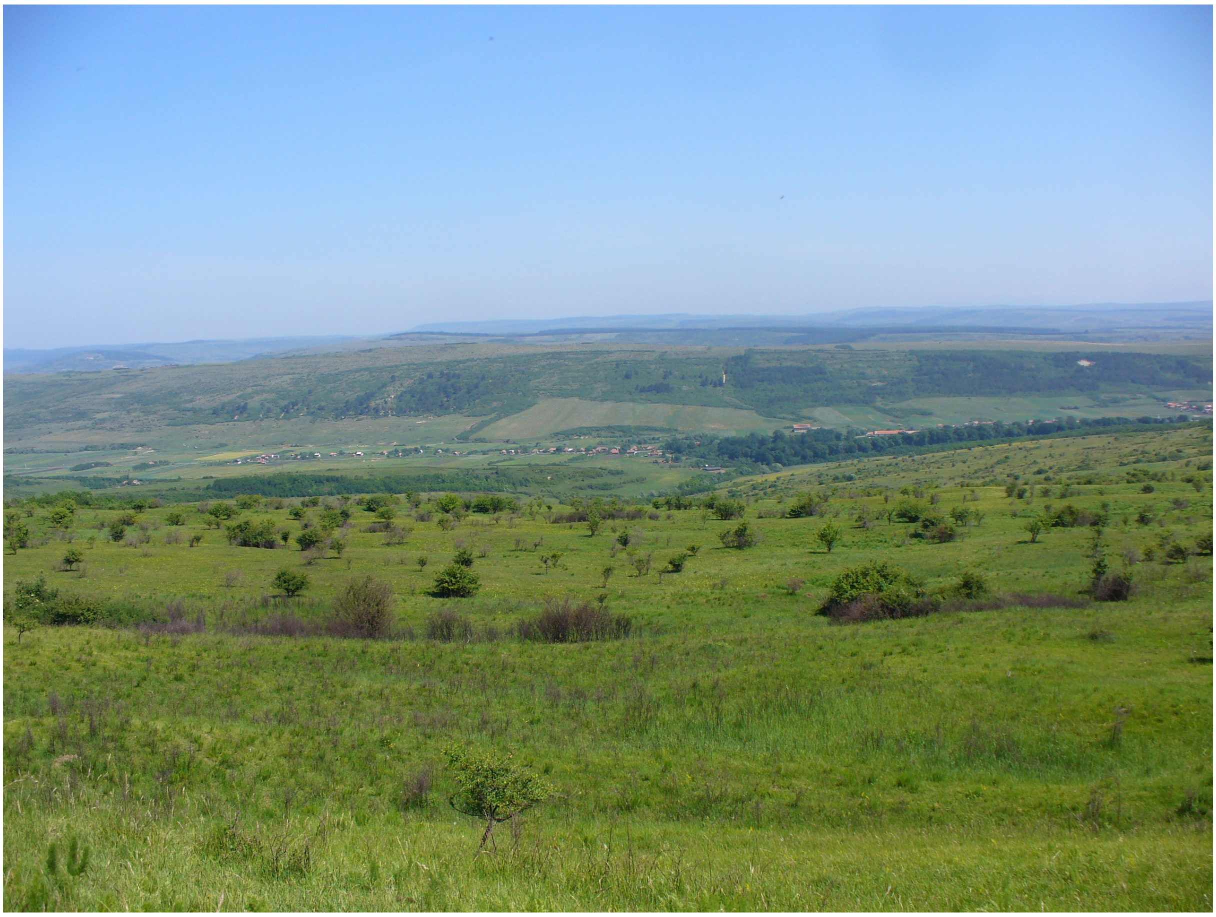

Supplement: Supplementary file 1 — Supplementary Information [file 41598_2017_12938_MOESM1_ESM.pdf]
